# The Effectiveness of Teamwork Training on Teamwork Behaviors and Team Performance: A Systematic Review and Meta-Analysis of Controlled Interventions

**DOI:** 10.1371/journal.pone.0169604

**Published:** 2017-01-13

**Authors:** Desmond McEwan, Geralyn R. Ruissen, Mark A. Eys, Bruno D. Zumbo, Mark R. Beauchamp

**Affiliations:** 1 School of Kinesiology, The University of British Columbia, Vancouver, British Columbia, Canada; 2 Departments of Kinesiology/Physical Education and Psychology, Wilfrid Laurier University, Waterloo, Ontario, Canada; 3 Department of Educational and Counseling Psychology, Faculty of Education, University of British Columbia, Vancouver, British Columbia, Canada; Rijksuniversiteit Groningen, NETHERLANDS

## Abstract

The objective of this study was to conduct a systematic review and meta-analysis of teamwork interventions that were carried out with the purpose of improving teamwork and team performance, using controlled experimental designs. A literature search returned 16,849 unique articles. The meta-analysis was ultimately conducted on 51 articles, comprising 72 (*k*) unique interventions, 194 effect sizes, and 8439 participants, using a random effects model. Positive and significant medium-sized effects were found for teamwork interventions on both teamwork and team performance. Moderator analyses were also conducted, which generally revealed positive and significant effects with respect to several sample, intervention, and measurement characteristics. Implications for effective teamwork interventions as well as considerations for future research are discussed.

## Introduction

From road construction crews and professional soccer squads to political parties and special operations corps, teams have become a ubiquitous part of today’s world. Bringing a group of highly-skilled individuals together is not sufficient for teams to be effective. Rather, team members need to be able to work well together in order for the team to successfully achieve its purposes [1, 2]. As a result, there has been a proliferation of research assessing whether, and how, teams can be improved through teamwork training. A wide range of studies have shown positive effects of teamwork interventions for improving team effectiveness across several contexts such as health care (e.g., [3]), military (e.g., [4]), aviation (e.g., [5]), and academic (e.g., [6]) settings. Similarly, improvements in teamwork have been observed as a result of training with a variety of team types including new teams (e.g., [7]), intact teams (e.g., [8]), and those created for laboratory-based experiments (e.g., [9]). In sum, the extant empirical evidence to date appears to suggest that teams can be improved via teamwork training.

### What is Teamwork?

Within teams, members’ behaviors can be categorized in terms of both *taskwork* and *teamwork* processes [2]. Marks et al. [10] differentiated between the two by suggesting that “taskwork represents *what* it is that teams are doing, whereas teamwork describes *how* they are doing it with each other” (p. 357). Specifically, while taskwork involves the execution of core technical competencies within a given domain, teamwork refers to the range of interactive and interdependent behavioral processes among team members that convert team inputs (e.g., member characteristics, organizational funding, team member composition) into outcomes (e.g., team performance, team member satisfaction) [2, 10]. Some examples of teamwork (and respective comparisons to taskwork) include: the seamless communication between a surgeon, nurse, and anaesthesiologist, rather than the technical competencies of these practitioners; the synergy between a quarterback and receiver to complete a passing play, rather than their respective skill sets related to throwing or catching a football; the collaborative adjustments a flight crew makes in response to adverse weather or system problems, rather than each individual’s aviation skills; and so forth. Research from an assortment of studies indicates that teamwork—the focus of the current paper—is positively related to important team effectiveness variables, including team performance, group cohesion, collective efficacy, and member satisfaction [1].

Teamwork has been conceptualized within several theoretical models. For example, in their review, Rousseau et al. [2] reported that 29 frameworks related to teamwork have been published. Although there is much overlap across these models, there are also some notable differences. These relate to the number of dimensions of teamwork being conceptualized as well as the specific labelling of these dimensions. One thing that is generally agreed upon, however, is that teamwork is comprised of multiple observable and measurable *behaviors*. For instance, two highly cited frameworks by Marks et al. [10] and Rousseau et al. [2] consist of 10 and 14 dimensions of teamwork, respectively. In general, teamwork models focus on behaviors that function to (a) regulate a team’s performance and/or (b) keep the team together. These two components coincide with the two respective processes that Kurt Lewin, the widely recognized father of group dynamics, originally proposed all groups to be involved in: *locomotion* and *maintenance* [11].

With regard to regulating team performance (i.e., locomotion), teamwork behaviors include those that occur (a) before/in preparation for team task performance, (b) during the execution of team performance, and (c) after completing the team task [2]. First, with regard to teamwork behaviors that occur *before/in preparation* for team task performance, these include the active process of defining the team’s overall purpose/mission, setting team goals, and formulating action plans/strategies for how goals and broader purposes will be achieved. These behaviors help ensure that all team members are clear in terms of what is required of them in order for the team to function effectively. Second, teamwork behaviors that occur *during the execution* of team tasks include actions that correspond to members’ communication, coordination, and cooperation with each other. At this stage, team members translate what they have previously planned (during the preparation phase) into action. Third, in terms of teamwork behaviors that occur *after completing* the team task (i.e., reflection), these include monitoring important situations and conducting post-task appraisals of the team’s performance and system variables (e.g., internal team resources, broader environmental conditions), solving problems that are precluding team goal attainment, making innovative adjustments to the team’s strategy, and providing/receiving verbal and behavioral assistance to/from teammates. Hence, team members determine whether their actions have moved them closer towards accomplishing the team goals and objectives, and whether any modifications are required in order to facilitate future success. In addition to these three dimensions concerned with the regulation of team performance, a fourth dimension of teamwork involves behaviors that function to keep the team together (i.e., maintenance). These behaviors focus on the team’s *interpersonal dynamics*, and include the management of interpersonal conflict between members and the provision of social support for members experiencing personal difficulties. Managing interpersonal dynamics is critical as it is theorized that teams cannot operate effectively when these issues are present [2].

### How Can Teamwork Be Trained?

Teamwork interventions have utilized a number of training methods in order to target the regulation of team performance (i.e., preparation, execution, reflection) and management of team maintenance (i.e., interpersonal dynamics) dimensions. These intervention strategies generally fall under one of four categories. First, the most basic approach to training and developing teamwork involves providing didactic education to team members in a classroom-type setting, such as lecturing about the importance of providing social support within the team or promoting ways to manage interpersonal conflict among teammates. This type of training has been found to be useful for enhancing team effectiveness (e.g., [12]). A second category of team training involves utilizing a more interactive workshop-style format, wherein team members take part in various group activities, such as having discussions about the team’s purposes and goals (e.g., [13]) or working through case studies together (e.g. [14]). The third broad category of team training involves simulation training, wherein teams experientially enact various teamwork skills, such as interpersonal communication and coordination, in an environment that mimics upcoming team tasks (e.g., airline simulators or medical patient manikins). Although often used as a means of fostering taskwork competencies (e.g., teaching new surgeons how to perform the technical skills of a medical operation), simulation training has been found to be an efficacious approach to teamwork intervention (e.g., [15]). In addition to these three training approaches that occur outside of the team task environment (i.e., training within classroom and simulation settings), teamwork can also be fostered by incorporating team reviews in-situ (i.e., where the team actually performs its tasks), which allows teams to monitor/review their quality of teamwork on an ongoing basis. These team reviews involve some form of team briefs before (e.g., creating action plans), during (e.g., monitoring team members’ actions), and/or after (e.g., assessing the team’s performance) team task execution, and have also been shown to be efficacious in previous studies (e.g., [16]).

The effectiveness of teamwork interventions can be determined with an assortment of criteria, including team- and individually-based behaviors, cognitions, and affective states. Hackman and Katz 2010 [17] posit that team effectiveness can be determined by examining the extent to which the team has achieved its a priori objectives. Since the broad purpose of forming a team is to produce something of value, it is perhaps unsurprising that the most widely tested criterion of team effectiveness has been team performance [18–20]. Thus, although teams come from an array of settings and are idiosyncratic in their own ways, one question that essentially all teams address at some point during their tenure is whether they are performing well. For example, is that road construction crew fixing potholes adequately? Does the local soccer squad have a respectable winning percentage? Has an elected political party successfully completed the tasks for which they campaigned? Did a special operations corps achieve the mission it set out to accomplish? When taken in concert, questions related to team performance are often of central interest when characterizing a team’s effectiveness.

In addition to assessing the outcome variable of team performance, researchers have also been interested in whether teamwork training actually improves teamwork itself. The efficacy of these interventions can be determined with a number of objective (e.g., products produced by an industry team), self-report (e.g., questionnaires regarding perceived social support amongst team members), and third-party assessments (e.g., expert ratings of team behaviors). Both general/omnibus measures of teamwork (e.g., [21]) as well as those assessing specific dimensions of teamwork (e.g., communication [22]) have been operationalized to examine the effectiveness of these interventions. For example, do team goal setting activities actually result in members creating and pursuing effective team goals? Does simulation training improve the requisite coordination processes among aviation cockpit crews? Has a didactic lecture contributed to improved conflict management among team members? Answering these types of questions is important for determining whether an intervention is actually efficacious in changing the variable that is targeted for improvement (i.e., teamwork behaviors).

### The Current Review

Prior to outlining the purposes of this systematic review, it is important to recognize that previous quantitative reviews have been conducted that addressed—to some degree—teamwork training. In preparation for this systematic review, we conducted a scoping review which revealed that eight previous meta-analyses have assessed teamwork intervention studies in some way. However, these reviews were delimited based on various sample and/or intervention characteristics. For example, some reviews included studies that were only conducted with certain team types (e.g., intact teams [23]) or within a particular context (e.g., sports [24]; medical teams [25]). Others were delimited to specific training programs/strategies that were restricted to a narrow range of teamwork strategies (e.g., [23, 25–29]). Finally, studies that used a combination of teamwork *and* taskwork intervention components have been systematically reviewed [30]; however, these types of interventions result in a limited ability to determine the extent to which the resulting effects were due to teamwork training versus taskwork training.

It should also be noted that all but one [23] of these previous reviews pooled together studies that included a control condition (i.e., wherein teams do not receive any type of teamwork training) and those that did not (as mentioned above, that study only analyzed the effects of certain teamwork strategies). This is an important consideration, as it has been suggested that controlled and uncontrolled studies should not be combined into the same meta-analysis due to differences in study quality (which is a major source of heterogeneity) and since stronger conclusions can be derived from controlled interventions compared to uncontrolled interventions (e.g., [31]). Therefore, while previous systematic reviews have provided valuable contributions to the teamwork literature, a systematic review that assesses the effects of controlled teamwork interventions across a range of contexts, team types, and involving those that targeted diverse dimensions of teamwork appears warranted. In doing so, a more comprehensive assessment of the efficacy of these teamwork interventions is provided, while also having the capacity to look at the potential moderating effects of various sample, intervention, and measurement characteristics. Moreover, by including only controlled studies, one is able to make stronger conclusions regarding the observed effects.

The overall purpose of this study was to better understand the utility of teamwork training for enhancing team effectiveness. Specifically, a meta-analysis was conducted on controlled studies (i.e., comparing teams who have received teamwork training with those who have not) that have examined the effects of teamwork interventions on teamwork processes and/or team performance. To better disentangle the effectiveness of these studies, we also sought to assess potential moderators of these main effects; that is, to determine whether there are certain conditions under which the independent variable of teamwork training more strongly (or weakly) causally influences the dependent variables of teamwork behaviors or team performance [32]. The specific moderators that we assessed included: (a) the team context/field of study, (b) the type of teams that were trained, (c) the primary type of intervention method employed, (d) the dimensions of teamwork that were targeted in the intervention, (e) the number of dimensions targeted, (f) the types of measures used to quantify the training effects, and (g) in studies where teamwork was assessed as an outcome variable, the dimensions of teamwork that were measured. It was hypothesized that teamwork training would have a positive and significant effect on both teamwork and team performance and that these effects would be evident across a range of the aforementioned sample, intervention, and measurement characteristics/conditions.

## Methods

### Literature Search

Searches for potential articles were conducted in the following databases: *PsycInfo*, *Medline*, *Cochrane Central Register of Controlled Trials*, *SportDiscus*, and *ProQuest Dissertations and Theses*. Hand searches were also conducted across thirteen journals that typically publish articles on group dynamics (e.g., *Group Dynamics*: *Theory*, *Research*, *and Practice*; *Small Group Research*, *Journal of Applied Psychology*; *Personnel Psychology*, *Human Factors*; *Academy of Management Journal*, *Journal of Sport & Exercise Psychology*). In each database and journal search, the following combination of search terms were used: (*team* OR *interprofessional OR interdisciplinary*) AND (*intervention* OR *training* OR *building* OR *simulation*) AND (*teamwork* OR *mission analysis* OR *goal specification* OR *goal setting* OR *planning* OR *strategy* OR *coordination* OR *cooperation* OR *communication* OR *information exchange* OR *information sharing* OR *monitoring* OR *problem solving* OR *backing up* OR *coaching* OR *innovation* OR *adaptability* OR *feedback* OR *support* OR *conflict management* OR *situation awareness* OR *confidence building* OR *affect management*). These terms were based on various models of teamwork that exist within the literature (see Rousseau et al. [2] for an overview of these models). An additional search was conducted within these databases and journals using the search terms (*TeamSTEPPS* OR *Crew Resource Management* OR *SBAR* [Situation-Background-Assessment-Recommendation]), as several articles in the initial search used these specific training programs. We also searched the reference sections of the articles from past teamwork training review papers as well as from articles that initially met inclusion criteria to determine if any additional articles could be retrieved. The searches were conducted in September 2015 and no time limits were placed on the search strategy. Each article was first subjected to title elimination, then abstract elimination, and finally full-text elimination.

### Eligibility Criteria

To be included in the meta-analysis, a study needed to examine the effects of teamwork training by comparing teams in an experimental condition (i.e., those who received teamwork training) with those in a control condition (i.e., where teams did not receive teamwork training). Cross-sectional/non-experimental studies were excluded, as were intervention studies that did not include a control condition. As this review was only concerned with teamwork interventions, studies that focused on training taskwork—whether independent of, or in addition to, a teamwork intervention—were excluded. For example, as previously mentioned, simulation-based training (SBT) has been used as a means of training individuals to perform technical skills and also to enhance teamwork. In order for a SBT intervention to be included in this meta-analysis, it had to be clear that only teamwork (not technical skills) was being targeted during training. In order to address our primary research question, the study had to provide data on at least one teamwork dimension and/or team performance. The study also needed to provide sufficient statistics to compute an effect size. In cases of insufficient data, corresponding authors were contacted for this information. The articles were delimited to those published in the English language.

### Data Analysis

Articles that met the aforementioned eligibility criteria were extracted for effect sizes and coded independently with respect to seven moderators by two of the authors (DM and GR). Interrater reliability for the coding of these moderators was over 90%, *kappa* (SE) = 0.80 (0.01). The moderators examined were based on a scoping review (the purpose of which included identifying pertinent characteristics that were commonly reported in previous teamwork intervention research), which was conducted in preparation for this systematic review. The moderators that were examined in this review included (1) the context within which an intervention was conducted (*health care*, *aviation*, *military*, *academia*, *industry*, or *laboratory experiment)*, (2) the type of team targeted (*intact* or *new*), (3) the primary training method applied to conduct the intervention (*didactic education*, *workshop*, *simulation*, or *team reviews*), (4) the dimension(s) of teamwork (*preparation*, *execution*, *reflection*, and/or *interpersonal dynamics*) targeted in the intervention as well as (5) the number of dimensions targeted (between one and four), (6) the type of measure used to derive effect sizes (*self-report*, *third party*, or *objective measures*), and—when teamwork was assessed as the criterion variable—(7) the specific dimension(s) of teamwork that were measured (*general*, *preparation*, *execution*, *reflection*, and *interpersonal dynamics*).

Once coded, data were entered into the software *Comprehensive Meta-Analysis*, *Version 2* [33] and analyzed as a random-effects model (DerSimonian and Laird approach). This type of model assumes that there is heterogeneity in the effect sizes across the included studies and is the appropriate model to use in social science research, as opposed to a fixed-effects model (which assumes that effect sizes do not vary from study to study) [34, 35]. Where possible, effect sizes for each study were derived from means, standard deviations, and sample sizes at baseline and post-intervention [34, 36]. If these statistics were not fully provided, they were supplemented with *F*-statistics, *t* scores, correlations, and *p*-values to compute the effect size. Each study was given a relative weight based on its precision, which is determined by the study’s sample size, standard error, and confidence interval (i.e., the more precise the data, the larger the relative study weight) [34].

In instances where a study provided data to calculate multiple effect sizes (such as when several measures of the criterion variable—teamwork or team performance—were examined), these effects were combined into one overall effect size statistic (i.e., a weighted average) for that study. This was done to ensure that those studies that had multiple measures of teamwork or team performance were not given greater weight compared to studies that only provided one effect size (i.e., only had one measure of performance or teamwork), which could potentially skew the overall results [34]. The exception to this was when articles reported the effects of more than one intervention (i.e., had multiple experimental conditions), each of which had a unique teamwork training protocol. In these cases, an effect size from each intervention was computed. Thus, these articles would contribute multiple effect sizes to the total number of comparisons within the meta-analysis. To correct for potential unit-of-analysis errors in these particular articles, the sample size of the control condition was divided by the number of within-study comparisons [31]. For example, if three different types of teamwork interventions were compared to one control condition (e.g., which had a sample size of 30 participants), the *n* of the control condition was divided by 3 (i.e., 30/3 = 10) when calculating the effect sizes of those interventions. Cohen’s *d* was used as the effect size metric to represent the standardized effect (i.e., the average magnitude of effectiveness) of teamwork interventions on teamwork and team performance [37]. Standard errors and 95% confidence intervals were computed to test for the accuracy of the standardized effects obtained.

To reduce heterogeneity and improve the interpretability of the results, we pooled studies into those that measured teamwork as its criterion variable and those that measured team performance. Pooling studies in this manner not only reduces heterogeneity but also allowed us to identify the extent to which teamwork interventions impact team performance and, separately, the extent to which they affect teamwork processes. Heterogeneity within the meta-analysis was also assessed by computing a *Q* value—which estimates the variability in the observed effect sizes across studies—and an *I*^2^ statistic—which estimates the ratio of the true heterogeneity to the total observed variation across studies. High *Q* and *I*^*2*^ statistics can be problematic for interpreting the results of a meta-analysis and can also indicate that the meta-analysis includes outlier studies. We also planned to identify and exclude outliers from subsequent moderator analyses in two ways. First, sensitivity analyses were carried out by removing a single intervention from the meta-analysis and noting the resulting effect size—this estimates the impact that each individual intervention has on the overall effect size of teamwork or team performance. If the resulting effect size with an intervention removed (i.e., K– 1) is substantially different than the effect size with that intervention present, this may suggest that it is an outlier and needs to be removed [34]. Second, we noted any studies that had abnormally high effect sizes and standardized residuals (above 3.0), especially when these values were accompanied by narrow confidence intervals. If heterogeneity (*Q* and *I*^2^) is substantially reduced upon removal of a study, this further confirms that the study is an outlier and should be omitted from subsequent subgroup/moderator analyses.

Once the two pools of studies were produced, bias within each pool was assessed. First, publication bias was examined by calculating a fail-safe *N* statistic, which estimates the number of unpublished studies with null findings that would have to exist to reduce the obtained effect size to zero [38]. If this number is sufficiently large—Rosenberg [39] recommends a critical value of 5*N*+10—then the probability of such a number of studies existing is considered to be low. For example, if 20 studies were included in a meta-analysis, then the resulting fail-safe *N* should be larger than 110 (i.e., 5*20 + 10); if this value was not larger than 110, then publication bias is likely within this pool of studies. We also obtained two funnel plots (one for studies where teamwork was the outcome variable and one for team performance as the outcome) to provide a visual depiction of potential publication bias. We then conducted an Egger’s test as a measure of symmetry for these two funnel plots. If this test statistic is significant (*p* < 0.05), this denotes that the distribution around the effect size is asymmetric and publication bias is likely present [34].

## Results

### Literature Search

**Fig 1:**
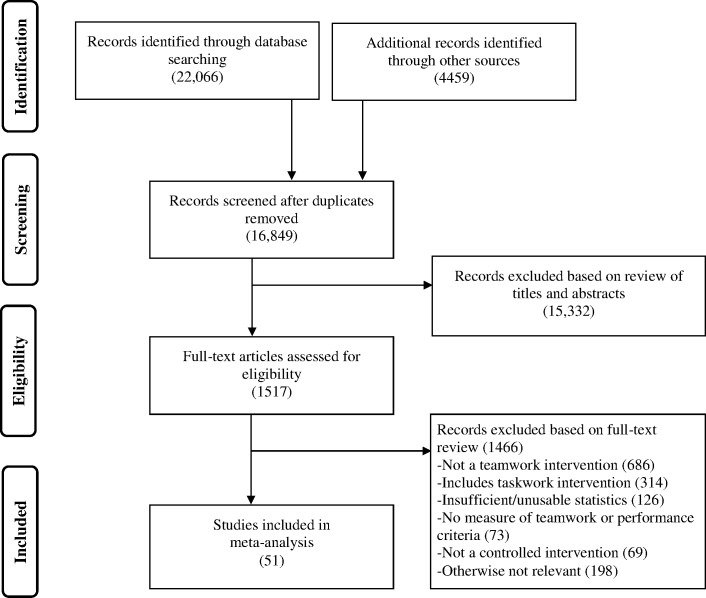
Results of Literature Search (PRISMA Flow Diagram).

The literature search from the five databases returned 22,066 articles, while the hand searches of the 13 journals returned 3797 articles, vetting of studies from previous team training reviews returned 191 articles, and the ancestry search of reference lists returned 471 articles (see Fig 1). After removing duplicates, 16,849 articles were subject to title and abstract screening, where they were dichotomously coded as ‘potentially relevant’ or ‘clearly not relevant’. 1517 potentially relevant articles were then full-text reviewed and coded as meeting eligibility criteria or as ineligible for the following reasons: (1) not a teamwork intervention; (2) teamwork-plus-taskwork intervention; (3) insufficient statistics to compute an effect size; (4) not including a measure of teamwork or team performance; or (5) not including a control group. As a result of this eligibility coding, 51 articles were included in the meta-analysis. 13 of these studies reported results on two or more interventions, bringing the total number of comparisons (*k*) to 72 with 8439 participants (4966 experimental, 3473 control). See S1 Table for descriptions of each study with regard to study context, type of team and participants, targeted teamwork dimensions of the intervention, number of effect sizes, the criteria measured, and an overview of the intervention.

### Summary Statistics

**Fig 2:**
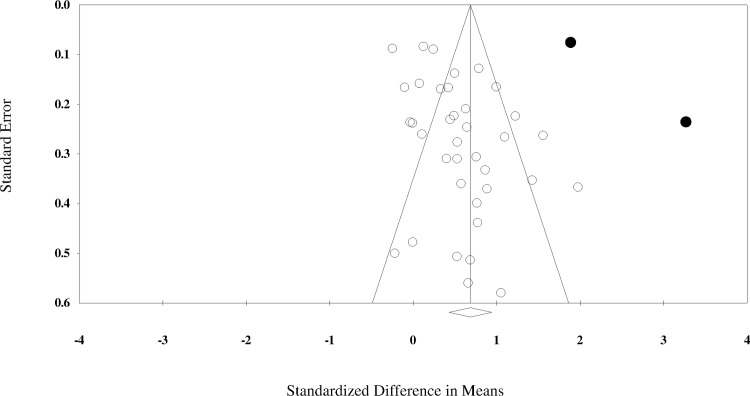
Funnel Plot for Studies Assessing Teamwork. Circles filled with black indicate outlier studies.

Results of the overall effect of teamwork interventions on teamwork processes along with summary statistics and sensitivity analyses (i.e., the final column marked ‘ES with study removed’) for this pool of studies are presented in Table 1. This pool included a total of 39 interventions from 33 studies. The results revealed that teamwork interventions had a significant, medium-to-large effect on teamwork, *d* (*SE*) = 0.683 (0.13), 95% *CI* = 0.43–0.94, *Z* = 5.23, *p* < 0.001; *Q* (*df*) = 660.7 (38), *I*^*2*^ = 94.2. The funnel plot for this pool of studies is shown in Fig 2. The fail-safe N was 3598, which is sufficiently large, as it exceeds the critical value of 205 (5*39+10). The funnel plot for this pool of studies is presented in Fig 2. Egger’s value for this funnel plot was not significant (*B* = 0.364, *SE* = 1.30, 95% *CI* = -2.26–2.99, *t* = 0.28, *p* = 0.78), which also suggests that bias was not present. Two studies were identified as outliers within this pool of studies: Morey et al. [3] and Marshall et al. [22]. The resulting effect size when these studies were excluded was *d* (SE) = 0.550 (0.08), 95% *CI* = 0.39–0.71, *Z* = 6.73, *p* < 0.001; *Q* (*df*) = 187.53 (36), *I*^*2*^ = 80.8. Subsequent moderator analyses were conducted with these two outlier studies being omitted.

**Table 1:** Summary Results of Interventions Assessing the Effects of Teamwork Training on Teamwork.

Study	Relative Weight	Effect Size (SE)	95% CI (lower, upper)	*Z*-value	*p*-value	ES with intervetion removed
Aaron 2014 [13] a	2.43	1.432 (.35)	.74, 2.13	4.04	< .001	0.67
b	2.48	.869 (.33)	.22, 1.52	2.61	.009	0.68
Becker 2005 [40]	2.75	.635 (.21)	.22, 1.05	3.02	.003	0.69
Beck-Jones 2004 [41] a	2.70	-.030 (.24)	-.50, .44	-0.13	.898	0.70
b	2.69	-.003 (.24)	-.47, .47	-0.01	.990	0.70
Beranek 2005 [42]	2.67	.649 (.25)	.16, 1.13	2.62	.009	0.68
Bjornberg 2014 [9]	2.83	.080 (.16)	-.23, .39	0.50	.615	0.69
Brannick 2005 [5]	2.72	1.229 (.23)	.79, 1.67	5.47	< .001	0.69
Bushe 1995 [43] a	2.53	.405 (.31)	-.20, 1.01	1.31	.192	0.69
b	2.53	.534 (.31)	-.08, 1.14	1.71	.086	0.69
Cheater 2005 [12]	2.82	.336 (.17)	.00, .67	1.97	.049	0.69
Clay-Willaims 2013 [44] a	2.04	.531 (.51)	-.46, 1.53	1.05	.296	0.69
b	2.06	-.213 (.50)	-1.20, .77	-0.43	.671	0.70
c	2.12	0.000 (.48)	-.94, .94	0.00	1.00	0.70
Dalenberg 2009 [45]	2.82	1.001 (.17)	.68, 1.33	6.02	< .001	0.67
Deneckere 2013 [46]	2.92	.129 (.09)	-.04, .29	1.52	.129	0.70
Dibble 2010 [47]	2.92	-.242 (.09)	-.42, -.07	-2.72	.007	0.71
Eden 1986 [48]	2.92	.427 (.09)	.07, .42	2.73	.006	0.70
Ellis 2005 [14]	2.88	.792 (.13)	.54, 1.05	6.14	< .001	0.68
Emmert 2011 [49]	2.54	.763 (.31)	.16, 1.36	2.48	.013	0.68
Entin 1999 [50]	2.32	.771 (.40)	-.01, 1.55	1.93	.054	0.68
Friedlander 1967 [51]	2.72	.495 (.22)	.06, .94	2.21	.027	0.69
Green 1994 [52] a	1.91	.665 (.56)	-.44, 1.76	1.19	.236	0.68
b	1.87	1.058 (.58)	-.08, 2.20	1.82	.069	0.68
Jankouskas 2010 [7]	2.22	.778 (.44)	-.08, 1.64	1.77	.077	0.68
Kim 2014 [53]	2.65	.062 (.26)	-.45, .57	0.24	.813	0.70
Marshall 2009 [22]*	2.70	3.277 (.33)	2.65, 3.95	9.90	< .001	0.61
Martinez-Moreno 2015 [54]	2.86	.503 (.14)	.23, .78	3.63	< .001	0.69
Morey 2002 [3]*	2.93	1.896 (.08)	1.75, 2.05	24.83	< .001	0.64
O’Leary 2011 [21]	2.82	.426 (.17)	.10, .76	2.54	.011	0.69
Padmo Putri 2012 [6]	2.82	-.097 (.17)	-.42, .23	-0.58	.561	0.71
Prichard 2007 [55]	2.40	1.981 (.37)	1.26, 2.70	5.381	< .001	0.65
Rapp 2007 [56]	2.61	.535 (.28)	-.01, 1.08	1.93	.053	0.69
Shapiro 2004 [57]	2.03	.689 (.52)	-.32, 1.70	1.34	.181	0.68
Smith-Jentsch 2008 [4]	2.63	1.103 (.27)	.58, 1.63	4.13	< .001	0.67
Thomas 2007 [58]	2.39	.891 (.37)	.16, 1.62	2.40	.016	0.68
Volpe 1996 [59]	2.71	.450 (.23)	.00, .90	1.97	.049	0.69
Weaver 2010 [60]	2.41	.580 (.36)	-.13, 1.29	1.61	.109	0.69
Weller 2014 [61]	2.64	1.563 (.26)	1.05, 2.08	5.92	< .001	0.66
**OVERALL**	**100**	**.683 (0.13)**	**0.43, 0.94**	**5.23**	**<0.001**	

*Note*. a, b, c = intervention groups within study; SE = standard error; CI = confidence interval; ES = effect size.

* = Study identified as an outlier and removed from subsequent moderator analyses.

The final column marked ‘ES with study removed’ indicates the results of the sensitivity analysis for each respective intervention.

**Fig 3:**
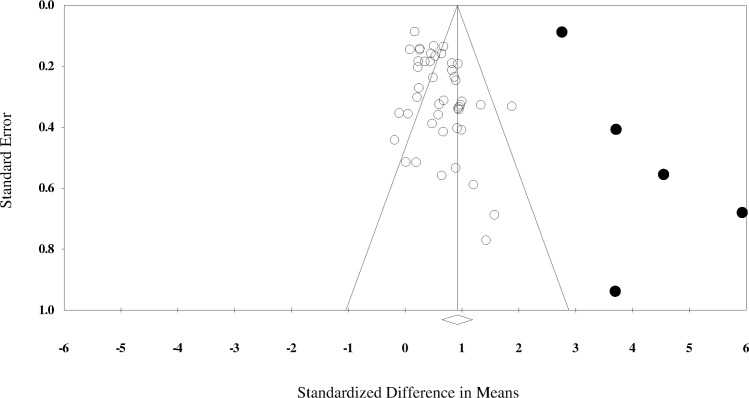
Funnel plot for studies assessing team performance. Circles filled with black indicate outlier studies.

Results of the overall effect of teamwork interventions on team performance as well as summary statistics and sensitivity analyses (i.e., the final column marked ‘ES with intervention removed’) for this pool of studies are presented in Table 2. This pool of studies included a total of 50 interventions from 32 studies. It was shown that teamwork interventions had a significant, large effect on team performance—*d* (*SE*) = 0.919 (0.14), 95% *CI* = 0.65–1.19, *Z* = 6.72, *p* < 0.001; *Q* (*df*) = 851.3 (49), *I*^*2*^ = 94.2. The funnel plot for this pool of studies is shown in Fig 3. The fail-safe N was 6692, which is sufficiently large, as it exceeds the critical value of 260 (5*50+10). The funnel plot for this pool of studies is presented in Fig 3. Egger’s value for this funnel plot was not significant (*B* = 0.131, *SE* = 1.19, 95% *CI* = -2.26–2.54, *t* = 0.11, *p* = 0.91), which also implies that bias was not present. There were five outlier interventions (from four studies) in this pool of studies that assessed team performance: Morey et al. [3], Smith-Jentsch et al. [4], one of the interventions from Buller and Bell [63]; teambuilding condition), and both interventions from Bushe and Coetzer [43]. When these outliers were removed, the resulting effect size was *d* (*SE*) = 0.582 (0.06), 95% *CI* = 0.47–0.69, *Z* = 10.30, *p* < 0.001; *Q* (*df*) = 101.1 (44), *I*^*2*^ = 56.5. Subsequent moderator analyses were conducted with these five interventions omitted.

**Table 2:** Summary Results of Interventions Assessing the Effects of Teamwork Training on Team Performance.

Study	Relative Weight	Effect Size (SE)	95% CI (lower, upper)	*Z*-value	*p*-value	ES with intervention removed
Beck-Jones 2004 [41] a	2.16	.502 (.18)	.35, 1.04	3.91	< .001	0.93
b	2.15	.902 (.18)	.33, 1.30	3.83	< .001	0.92
Bjornberg 2014 [9]	2.24	.466 (.16)	.15, .78	2.91	.004	0.93
Brannick 2005 [5]	2.20	.237 (.21)	-.17, .64	1.15	.249	0.94
Brown 2003 [62]	2.25	.267 (.15)	-.02, .56	1.80	.072	0.94
Buller 1986 [63] a	1.33	1.435 (.77)	-0.08, 2.95	1.86	.063	0.91
b*	1.11	3.72 (.94)	1.88, 5.56	3.96	< .001	0.89
C	1.46	1.58 (.69)	.23, 2.94	2.30	.022	0.91
Bushe 1995 [43] a*	1.67	4.57 (.56)	3.47, 5.66	8.19	< .001	0.86
b*	1.47	5.96 (.68)	4.63, 7.29	8.75	< .001	0.84
Cannon-Bowers 1998 [64]	2.22	.46 (.19)	.09, .82	2.45	.014	0.93
Chang 2008 [65]	2.04	1.344 (.33)	.70, 1.99	4.09	< .001	0.91
Dalenberg 2009 [45]	2.24	.653 (.16)	.34, .97	4.06	< .001	0.93
Dibble 2010 [47]	2.29	.181 (.09)	.01, .36	2.04	.042	0.94
Entin 1999 [50]	1.92	.927 (.41)	.13, 1.72	2.88	.022	0.92
Fandt 1990 [66]	2.25	.095 (.15)	-.19, .38	0.65	.518	0.94
Green 1994 [52] a	1.67	.655 (.56)	-.44, 1.75	1.17	.243	0.92
b	1.62	1.212 (.59)	.05, 2.37	2.05	.040	0.91
Haslam 2009–1 [67] a	2.08	.223 (.30)	-.37, .82	0.73	.464	0.93
b	2.06	.690 (.31)	.07, 1.31	2.20	.028	0.92
Haslam 2009–2 [67] a	2.02	.941 (.34)	.27, 1.61	2.76	.006	0.92
b	2.04	.610 (.33)	-.03, 1.25	1.87	.062	0.93
c	2.02	.957 (.35)	.28, 1.63	2.78	.005	0.92
d	2.03	.963 (.34)	.31, 1.62	2.87	.004	0.92
Ikomi 1999 [68]	2.06	1.008 (.32)	.39, 1.63	3.18	.001	0.92
Jankouskas 2010 [7]	1.86	-.173 (.44)	-1.04, .70	-0.39	.696	0.94
Jarrett 2012 [69] a	2.22	.243 (.19)	-.12, .61	1.31	.191	0.94
b	2.21	.834 (.19)	.46, 1.21	4.34	< .001	0.92
c	2.22	.358 (.19)	-.01, .72	1.92	.055	0.93
d	2.21	.940 (.19)	.56, 1.32	4.84	< .001	0.92
Kring 2005 [70] a	2.00	.062 (.36)	-.64, .76	0.17	.862	0.94
b	2.00	-.092 (.36)	-.79, .61	-0.26	.795	0.94
Longenecker 1994 [71]	2.03	1.89 (.33)	1.24, 2.54	5.66	< .001	0.90
Morey 2002 [3]*	2.29	2.781 (.09)	2.61, 2.95	31.51	< .001	0.80
Padmo Putri 2012 [6]	2.23	.542 (.17)	.21, .87	3.21	.001	0.93
Rapp 2007 [56]	2.12	.254 (.27)	-.28, .79	0.93	.353	0.93
Schurig 2013 [72] a	2.26	.513 (.27)	-.02, 1.05	1.88	.061	0.93
b	2.26	.688 (.28)	.15, 1.23	2.49	.013	0.93
Siegel 1973 [73]	1.99	.594 (.36)	-.11, 1.30	1.64	.100	0.93
Sikorski 2012 [74]	2.26	.272 (.14)	-.01, .56	1.89	.059	0.94
Smith-Jentsch 2008 [4]*	1.91	3.729 (.41)	2.92, 4.54	9.07	< .001	0.86
Smith-Jentsch 1996 [75] a	1.74	.206 (.52)	-.81, 1.22	0.40	.690	0.93
b	1.74	.025 (.52)	-.99, 1.04	0.05	.961	0.94
c	1.71	.901 (.54)	-.15, 1.95	1.68	.092	0.92
Stout 1997 [76]	2.04	.984 (.33)	.34, 1.63	3.00	.003	0.92
Villado 2013 [16]	2.19	.834 (.22)	.41, 1.36	3.88	< .001	0.92
Volpe 1996 [59]	2.16	.877 (.24)	.28, 1.12	3.70	< .001	0.92
Wegge 2005 [77] a	1.91	1.004 (.41)	.19, 1.81	2.44	.015	0.92
b	1.90	.682 (.42)	-.14, 1.50	1.64	.102	0.92
c	1.95	.487 (.39)	-.28, 1.25	1.25	.212	0.93
**OVERALL**	**100**	.919 (.14)	.65, 1.19	6.72	**<0.001**	

*Note*. a, b, c, d = intervention groups within study; SE = standard error; CI = confidence interval; ES = effect size.

* = Study identified as an outlier and removed from subsequent moderator analyses.

The final column marked ‘ES with study removed’ indicates the results of the sensitivity analysis for each respective intervention.

### Moderator Analyses

The results of the moderator analyses are shown in Table 3 (for teamwork behaviors) and Table 4 (for team performance). With respect to sample characteristics, significant positive effects of teamwork interventions were found for enhancing teamwork across all contexts (*d*s = 0.46–1.23) except for the single effect size from an industry setting (*d* = 0.50). In terms of team performance, significant effects were evident across all settings (*d*s = 0.40–1.76). In addition, interventions were effective for enhancing teamwork with intact teams (*d* = 0.33) and newly-formed teams (*d* = 0.67), with the effect size for new teams being significantly larger (*Q* = 4.04, *p* = 0.004) than that for existing teams. Teamwork training was also effective at fostering team performance for both team types; however, in contrast to the findings on teamwork, the effect size for intact teams (*d* = 0.99) was significantly larger (*Q* = 6.04, *p* = 0.02) than that for new teams (*d* = 0.54).

**Table 3:** Moderator results for interventions assessing teamwork as the outcome variable.

Moderator	K	Effect size (SE)		95% CI	Z-value	p value	*Q* value (df), *p*-value
**Sample Characteristics**							
Context							3.272(5), *p* = 0.658
Health care	13	0.51 (0.15)		0.20, 0.81	3.30	0.001	
Academia	10	0.46 (0.17)		0.14, 0.78	2.78	0.005	
Laboratory experiment	6	0.51 (0.20)		0.12, 0.89	2.55	0.011	
Military	6	0.77 (0.23)		0.33, 1.22	3.42	0.001	
Aviation	1	1.23 (0.47)		0.25, 2.21	2.46	0.014	
Industry	1	0.50 (0.50)		-0.48, 1.47	0.99	0.321	
Team type							4.04(1), *p* = 0.004
Intact	13	0.33 (0.14)		0.05, 0.60	2.35	0.019	
New	24	0.67 (0.10)		0.47, 0.87	6.58	<0.001	
**Intervention Characteristics**							
Method of intervention							6.17(3), *p* = 0.10
Didactic education	4	0.19 (0.19)		-0.20, 0.57	0.95	0.341	
Workshop	18	0.50 (0.10)		0.31, 0.70	4.96	<0.001	
Simulation	11	0.78 (0.16)		0.48, 1.09	5.05	<0.001	
Team Reviews	4	0.64 (0.19)		0.26, 1.01	3.34	0.001	
Teamwork dimensions targeted ^a^							
Preparation	20	0.75 (0.11)		0.54, 0.95	7.09	<0.001	
Execution	21	0.64 (0.11)		0.42, 0.86	5.70	<0.001	
Reflection	22	0.65 (0.11)		0.43, 0.86	5.80	<0.001	
Interpersonal dynamics	11	0.69 (0.16)		0.38, 1.00	4.33	<0.001	
Number of dimensions targeted ^b^							19.73(4), *p* = 0.001
One	6		0.05 (0.16)	-0.26, 0.35	0.29	0.775	
Two	11		0.65 (0.12)	0.42, 0.89	5.39	<0.001	
Three	6		0.98 (0.16)	0.66, 1.30	6.04	<0.001	
Four	7		0.57 (0.15)	0.27, 0.87	3.70	<0.001	
**Measurement Characteristics**							
Type of teamwork measure ^c^							16.86(1), *p*<0.001
Third party	45	0.80 (0.07)		0.66, 0.94	10.92	<0.001	
Self-report	46	0.38 (0.07)		0.25, 0.52	5.47	<0.001	
Teamwork dimension measured ^c^							2.98(1), *p* = 0.56
General	27	0.71 (0.11)		0.49, 0.93	6.36	<0.001	
Preparation	8	0.53 (0.19)		0.16, 0.89	2.80	0.005	
Execution	31	0.55 (0.10)		0.35, 0.74	5.57	<0.001	
Reflection	12	0.70 (0.16)		0.40, 1.01	4.50	<0.001	
Interpersonal dynamics	13	0.45 (0.14)		0.17, 0.73	3.12	0.002	

*Note*. The df of the Q-value represents the total number of combinations of the targeted dimensions minus 1.

^a^: The total k of this moderator is greater than 37 as many interventions targeted more than one dimension of teamwork. Because of this, each category within this moderator was analyzed independently (i.e., whether each teamwork dimension was targeted or not targeted in the intervention); as a result, it was not possible to calculate a *Q* value for this moderator.

^b^: The total k of this moderator is less than 37 as seven interventions were unclear in terms of the exact teamwork dimensions targeted.

^c^: The total k of this moderator is greater than 37 as many studies used more than one type of criterion measure of teamwork. Because of this, each category within this moderator was analyzed independently.

**Table 4:** Moderator results for interventions assessing team performance as the outcome variable.

Moderator	k	Effect size (SE)	95% CI	Z value	p value	Q value (df), *p*-value
**Sample Characteristics**						
Context						16.94(5), *p* = 0.01
Health care	2	0.76 (0.31)	0.15, 1.36	2.46	0.014	
Laboratory experiment	25	0.54 (0.07)	0.41, 0.67	8.08	<0.001	
Aviation	4	0.64 (0.18)	0.28, 0.99	3.51	<0.001	
Military	5	0.66 (0.17)	0.34, 0.99	3.99	<0.001	
Industry	3	1.76 (.32)	1.13, 2.38	5.52	<0.001	
Academia	6	0.40 (0.12)	0.17, 0.63	3.35	0.001	
Team type						6.04(1), *p* = 0.02
Intact	6	0.99 (0.18)	0.64, 1.33	5.63	<0.001	
New	39	0.54 (0.06)	0.42, 0.65	9.32	<0.001	
**Intervention Characteristics**						
Method of intervention						2.44(3), *p* = 0.49
Didactic education	4	0.41 (0.16)	0.09, 0.74	2.52	0.012	
Workshop	24	0.55 (0.08)	0.39, 0.71	6.87	<0.001	
Simulation	7	0.57 (0.17)	0.23, 0.90	3.30	0.001	
Team Reviews	10	0.69 (0.10)	0.50, 0.89	6.88	<0.001	
Teamwork dimensions targeted ^a^						
Preparation	15	0.60 (0.07)	0.46, 0.73	8.69	<0.001	
Execution	26	0.52 (0.08)	0.37, 0.66	6.87	<0.001	
Reflection	22	0.55 (0.08)	0.40, 0.70	7.17	<0.001	
Interpersonal dynamics	6	0.57 (0.18)	0.18, 0.95	2.88	0.004	
Number of dimensions targeted ^b^						3.98(4), *p* = 0.67
One	20	0.61 (0.09)	0.44, 0.79	6.85	<0.001	
Two	12	0.63 (0.12)	0.40, 0.86	5.31	<0.001	
Three	9	0.46 (0.11)	0.24, 0.67	4.08	<0.001	
Four	3	0.67 (0.25)	0.19, 1.15	2.74	0.006	
**Measurement Characteristics**						
Type of team performance measure ^c^						2.03(1), *p* = 0.15
Third party	31	0.56 (0.08)	0.40, 0.72	6.79	<0.001	
Objective	62	0.61 (0.06)	0.48, 0.73	9.70	<0.001	

*Note*. The df of the Q-value represents the total number of combinations of the targeted dimensions minus 1.

^a^: The total k of this moderator is greater than 45 as many interventions targeted more than one dimension of teamwork. Because of this, each category within this moderator was analyzed independently (i.e., whether each teamwork dimension was targeted or not targeted in the intervention); as a result, it was not possible to calculate a *Q* value for this moderator.

^b^: The total k of this moderator is less than 45 as one intervention was unclear in terms of the exact teamwork dimensions targeted.

^c^: The total k of this moderator is greater than 45 as many studies used more than one type of criterion measure of team performance. Because of this, each category within this moderator was analyzed independently.

Three intervention characteristics were analyzed as potential moderators. First, with regard to the intervention method utilized, significant effects on teamwork were found for workshop training (*d* = 0.50), simulation-based teamwork training (*d* = 0.78), and team reviews (*d* = 0.64) but not for didactic education (*d* = 0.19). All training methods were effective for enhancing team performance (*d*s = 0.41–0.69). Second, significant effects of training on teamwork were evident when two or more dimensions of teamwork were targeted (*d*s = 0.65–0.98) but not when only one dimension was targeted (*d* = 0.05). Team performance, however, improved significantly as a result of teamwork training regardless of the number of teamwork dimensions that were targeted (*d*s = 0.46–0.67). Third, significant effects were shown regardless of which dimension (i.e., preparation, execution, reflection, interpersonal dynamics) was targeted for both teamwork (*d*s = 0.64–0.75) and team performance (*d*s = 0.52–0.60).

With regard to measurement characteristics, significant improvements on teamwork emerged when either third-party (*d* = 0.80) or self-report (*d* = 0.38) measures of teamwork were utilized; the effect size for third-party measures was significantly larger (*Q* = 6.02, *p* = 0.014) than the effect size for self-report measures. For team performance outcomes, significant effects were shown for both objective (*d* = 0.61) and third-party measures (*d* = 0.56). Finally, significant effects on teamwork were found when general/omnibus measures of teamwork were taken (*d* = 0.71), as well as when a specific dimension of teamwork was measured (*d*s = 0.45–0.70).

## Discussion

The purpose of this systematic review and meta-analysis was to quantify the effects of the extant controlled experimental research of teamwork training interventions on teamwork and team performance. We found positive and significant medium-to-large sized effects for these interventions on teamwork and large effects on team performance. When outlier studies were removed, medium-sized effects were found for both criteria. Additional subgroup/moderator analyses also revealed several notable findings, each of which will be discussed in turn. The paper concludes with a discussion of the limitations associated with this meta-analysis as well as considerations for future teamwork training research.

### Who Can Benefit From Teamwork Training?

With regard to sample characteristics, teamwork interventions were shown to be effective at enhancing both teamwork and team performance across a variety of team contexts, including laboratory settings as well as real-world contexts of health care, aviation, military, and academia. This highlights the efficacy of teamwork training as a means of improving teams; this is an important finding as effective teams (i.e., those that work well together and perform at a high level) are vital in many of the aforementioned contexts. For example, it has been estimated that approximately 70% of adverse events in medical settings are not due to individuals’ technical errors but, rather, as a result of breakdowns in teamwork [78]. Thus, there is a critical need to ensure that teams are effective across these settings, as these teams greatly impact (among other things) the welfare of others. The results of this meta-analysis suggest that teamwork training can indeed be a useful way of enhancing team effectiveness within these contexts.

We also examined whether there were differential effects of teamwork training for new teams compared to intact teams. It was shown that these interventions were effective for both team types. The effects of teamwork training on teamwork outcomes were significantly larger for new teams (who showed a medium-to-large effect size) compared to existing teams (who had a small-to-medium effect size). Interestingly, when we examined team performance as the criterion variable, the training effects were significantly larger for intact teams (who showed a large effect size) compared to newly-formed teams (who again showed a medium-to-large effect size). It should be noted that there were many more studies conducted with new teams compared to intact teams—thus, caution should be exercised in directly comparing these findings. Nonetheless, at this point, the existing research seems to suggest that teamwork interventions work particularly well at enhancing teamwork processes for newly established teams—and also work with existing teams—but not the same extent. It is possible that teamwork processes might be more malleable and display greater potential for improvement with new teams compared to more established teams whose teamwork processes may be more entrenched. On the other hand, it is notable that the effects of teamwork training on team performance were stronger for established teams. In line with this, it is plausible that, while intact teams may show less pronounced changes in teamwork, they might be better able to translate their teamwork training into improved team performance outcomes.

### What Type of Training Works?

Three moderator variables were assessed with regard to intervention characteristics. First, with regard to the training method utilized, it was shown that all four training methods were effective for enhancing team performance. These included the provision of didactic lectures/presentations, workshops, simulation training, and review-type activities conducted in situ. Although significant effects were shown for the latter three training methods for teamwork outcomes, those interventions that targeted didactic instruction did not result in significant improvements in teamwork itself. This suggests that simply providing educational lectures wherein team members passively learn about teamwork is not an effective way of improving teamwork. When taken together these findings suggest that teamwork training should incorporate experiential activities that provide participants with more active ways of learning and practising teamwork. These may include various workshop-style exercises that involve all team members, such as working through case studies of how teams can improve teamwork, watching and critiquing video vignettes of teams displaying optimal versus suboptimal teamwork, discussing and setting teamwork-related goals and action plans, or other activities that help stimulate critical thinking and active learning of effective teamwork. Teams may also find it useful to conduct simulations of specific team tasks that the group is likely to encounter in-situ, such as aviation teams using an airplane simulator, surgical teams conducting mock-surgeries on medical manikins, military teams practising various field missions, and so on. Teamwork can be also fostered by having team members participate in team reviews/briefings before, during, and/or after the execution of team tasks that occur in-situ. In summary, simply lecturing about the importance of teamwork is not sufficient to create meaningful improvements in teamwork; rather, substantive positive effects can be derived by having team members engage in activities that require them to *actively learn about* and *practise* teamwork.

We also sought to assess how comprehensive an intervention should be—specifically, the number of teamwork dimensions that need to be targeted—in order to be effective. With regard to improving team performance, there were significant effects when one or more dimensions were targeted. However, in terms of improving teamwork behaviors, significant effects only emerged when two or more dimensions were targeted. From an applied perspective, individuals concerned with intervention (e.g., team consultants, coaches, managers, team leaders) can utilize these findings by targeting more than one dimension of teamwork within their training protocol. For instance, if the purpose of an intervention is to improve a health care team’s communication, greater effects may be derived by not merely targeting communication during the execution phase alone (e.g., with a structured communication tool), but by also incorporating strategies that target other dimensions of teamwork, such as setting goals and action plans for how communication will be improved (i.e., the preparation dimension of teamwork) as well as monitoring progress towards those goals, resolving any communication-related problems that arise, and making adjustments to action plans as necessary (i.e., the reflection dimension).

Relatedly, we sought to address whether there were differential effects of teamwork interventions on teamwork and team performance based on the dimensions of teamwork that were targeted. It was found that interventions had a significant effect on both teamwork behaviors and team performance when any dimension of teamwork was targeted. This is important as it means that if those concerned with intervention target any one of the four dimensions of teamwork, this will likely result in improvements in team functioning. While the preparation (i.e., behaviors occurring before team task performance such as setting goals and action plans), execution (i.e., intra-task behaviors such as communication and coordination), and reflection (i.e., behaviors occurring following task performance such as performance monitoring and problem solving) dimensions have each been theorized to be implicated in fostering team performance [2, 79], is particularly noteworthy that interventions targeting the interpersonal dynamics of a team (i.e., managing interpersonal conflict and the provision of social support between members) also displayed significant effects in relation to team performance. Specifically, efforts to enhance interpersonal processes have generally been theorized to be related to supporting team maintenance more so than supporting team performance [2, 79]. However, the results from the current review provide evidence that training teams with regard to social support and interpersonal conflict management processes may actually be a useful way to enhance team performance. While the exact reason for this effect is not immediately clear from this review, it may be that improving interpersonal dynamics has an indirect relationship with team performance. That is, teamwork training focused on improving social support and conflict management may improve the functioning of a team, which, in turn, improves the team’s performance. As Marks et al. [10] contend, these interpersonal processes “lay the foundation for the effectiveness of other processes” (p. 368). Relatedly, Rousseau et al. [2] suggest that problems related to social support and conflict management “may prevent team members from fully contributing to task accomplishment or from effectively regulating team performance” (p. 557). Further research examining this potential relationship is required as this would have implications in both research and applied teamwork settings.

### Does It Matter How Criterion Variables Are Measured?

Two measurement characteristics were examined as moderators within this meta-analysis. First, significant, large- and small-to-medium sized effects were found for third party and self-report measures of teamwork, respectively. Significant medium effects were also evident for third party and objective measures of team performance. It is worth noting that significantly larger effect sizes emerged for third party assessments of teamwork compared to self-report measures. Taken together, these findings suggest that the positive effects that were found for teamwork interventions are not merely perceptive and/or due to individuals’ self-report biases (i.e., social desirability). Rather, these results indicate that the effects of these interventions on both teamwork and team performance are clearly observable with measures beyond self-report indices.

Finally, we sought to assess whether the effects of teamwork training varied based on which teamwork dimension(s) were measured. Medium-to-large effects emerged when general/omnibus measures of teamwork—that is, those that provided an overall score of teamwork as opposed to examining individual dimensions of teamwork—were taken. Measures that tapped into the specific dimensions of teamwork (e.g., those that provided individual scores on preparation, execution, reflection, and interpersonal dynamics) also yielded comparable effect sizes. Hence, teamwork interventions appear to have a somewhat similar effect on each of the components of teamwork. In summary, the results of the above two moderators (i.e., type of measure and dimension of teamwork examined) suggest that teamwork training has a positive impact on teamwork and team performance regardless of the way in which these variables are assessed.

### Limitations

Despite the contributions of this meta-analytic review, it is not without limitations. First, there were additional variables that we had planned to analyze as moderators *a priori* including team size and length of/contact time within the intervention. However, there was an insufficient amount of reliable data across the studies on these variables to conduct these subgroup analyses appropriately. For instance, although many studies noted the total number of participants within an organization (e.g., a hospital) that took part in an intervention, information on the size of the teams within the organization (e.g., various units within the hospital) was often missing. Team composition variables such as this have been noted as important factors to take into account when examining teams (e.g., [30, 80]). Similarly, although some studies were explicit about the total length of the intervention and the contact time between interventionists and participating teams, this information was not provided consistently. This too would have been a valuable feature to analyze in order to provide more specific recommendations about how teamwork training programs should be designed—that is, how long an intervention should last? Unfortunately, due to the paucity of information available in the included manuscripts, we were unable to determine whether these variables moderated the observed effects of teamwork training on teamwork and team performance in the current meta-analysis.

Furthermore, there was a considerable amount of variability within some of the moderator categories that were coded. For instance, with regard to intervention methods, ‘workshops’ consisted of many different types of activities including team charter sessions, strategy planning meetings, case study activities, and so on. Combining these activities into one category was done for the sake of being adequately powered to conduct moderator analyses (i.e., include a sufficient number of studies within each of the resulting categories). However, while the above examples are indeed activities that teams do together, they are of course each different in their own ways. Hence, although it is evident that workshop-type activities are effective overall, it is unclear if specific workshop activities are more effective than others. This example underscores the difficulty that can occur when trying to balance statistical power with accuracy for each moderator category when conducting subgroup analyses in a meta-analysis.

Relatedly, effect sizes were only computed with the statistics that were provided from baseline and post-intervention, even if studies provided additional data on teamwork and/or performance at some other point in between or at a follow-up point in time (although it is worth noting that relatively few studies actually did this). This was done in order to minimize heterogeneity within the meta-analysis and improve the interpretability of the results (i.e., determining the effects of teamwork training from pre- to post-intervention). However, by not taking these measurement time-points into consideration, two questions in particular are raised. First, do certain dimensions of teamwork and team performance evolve differently over time and, if so, how? For instance, do improvements in teamwork occur immediately in response to training and then plateau; or do they improve in a slower, more linear fashion from the onset of training? Second, what are the long-term implications of teamwork training? That is, does teamwork training result in sustained improvements in teamwork and team performance beyond the intervention period or do these effects eventually wane? Answers to these types of research questions would certainly be of interest to teamwork researchers and applied practitioners.

### Future Directions

In addition to summarizing the previous research on teamwork interventions for improving teamwork and team performance, the findings from this systematic review also highlight several potential avenues of future research. First, with regard to sample characteristics, the majority of studies that examined the effects of teamwork interventions on team performance were conducted within laboratory settings, with relatively fewer controlled studies having been conducted in real-world settings. Thus, although significant effects on team performance (and teamwork) were found in health care, aviation, military, and academic settings, the extant literature would be strengthened by conducting further controlled intervention research within these contexts. It was also shown that teamwork training was less effective for improving teamwork for intact teams compared to new teams. Since many teams seeking teamwork training are likely to be intact, it is important that future research continue to test various training strategies that can be utilized with these types of teams. In addition, there are other contexts in which controlled interventions have not yet been conducted such as with police squads, firefighting crews, sports teams, political parties, and so on. Research in these areas is clearly ripe for future inquiry.

Further research on the ideal *combination* of teamwork dimensions (i.e., preparation and/or execution and/or reflection and/or interpersonal dynamics) targeted in an intervention would also enhance our current knowledge in terms of how to train teamwork most effectively and efficiently. We had originally planned to further assess this moderator by conducting a method co-occurrence analysis [81]. Specifically, since there would likely be a variety of combinations of dimensions that were targeted in the teamwork interventions (e.g., preparation only; preparation and execution; preparation, execution, reflection, and interpersonal dynamics; etc), we had hoped to examine if there would be differential effects of these combinations with regard to intervention effectiveness. Unfortunately, since there were such a large number of combinations of dimensions targeted in the included studies, there was an insufficient number of interventions that fell into each category. We were, therefore, unable to pursue this method co-occurrence analysis [81] of the various combinations of dimensions. Thus, although our findings suggest that interventions are more effective when two or more dimensions are targeted, further research that examines the effects of the ideal *combinations* of these dimensions would certainly enhance our current knowledge of teamwork training. For example, if the objective of teamwork training is to improve the coordination and cooperation of the team, should the training also target (in addition to targeting these execution behaviors) *both* the preparation and reflection dimensions of training (or simply one or the other)? Answering such complex questions will help to advance our understanding of what makes for an effective teamwork training program.

## Conclusion

Balanced against the contributions and insights provided by the various moderator analyses conducted in this study, the overall take-home message is that teamwork training is an effective way to foster teamwork and team performance. These effects appear to be evident across a range of samples, utilizing numerous intervention methods, and when considering various measurement characteristics. Interventions appear to be particularly effective when they target multiple dimensions of teamwork and include experiential activities for team members to actively learn about, practise, and continually develop teamwork.

## Supporting Information

S1 Table Summaries of Interventions. Summaries of each study and intervention included in the meta-analysis is provided in the S1 Table.
(DOCX)

S1 File PRISMA Checklist. The Preferred Reporting Items for Systematic Reviews and Meta-Analyses (PRISMA) Checklist [82] for this review is presented in the S1 File.
(DOC)
