# Supplementary material for: The Effectiveness of Teamwork Training on Teamwork Behaviors and Team Performance: A Systematic Review and Meta-Analysis of Controlled Interventions
Source: PLoS One. 2017 Jan 13;12(1):e0169604. doi: 10.1371/journal.pone.0169604 (PMC5234826; doi:10.1371/journal.pone.0169604)
Supplement: S1 Table — Summaries of each study and intervention included in the meta-analysis is provided in the S1 Table. (DOCX) [file pone.0169604.s001.docx]

# S1 Table. Summaries of interventions.

| **Study (First author)** | **Team Type & Participants** | **Targeted Teamwork Dimension(s)** | **Effect Sizes and Criterion Measures** | **Description of Intervention** |
| --- | --- | --- | --- | --- |
| **HEALTH CARE** | |  |  |  |
| Chang 2008 [65] | New: medical students, office staff without clinical experience, and junior surgical residents forming 24 teams (12 intervention, 12 control); 2 members per team | Reflection (feedback) | 5: performance | **Experimental**: Teams received verbal feedback from experienced instructor on team task performance. **Control**: Teams received little or no verbal feedback on team task performance. |
| Cheater 2005 [12] | Intact: 22 teams within five hospitals (11 intervention teams, 77 members; 11 control teams, 64 members) | General teamwork intervention | 1: general execution | **Experimental**: Seven staff members from the five hospitals first took part in a two-day facilitation workshop focused on their roles as facilitator, values clarification and individual learning styles, team building and group working skills, dealing with conflict, project management, and an introduction to the intervention programme. The programme consisted of five 1.5-to-2-hour meetings over the following six months. **Control**: No training. |
| Clay-Williams 2013 [44] | Intact: doctors, nurses, and midwives from five hospitals assigned to classroom-only intervention (12 participants), simulation-only intervention (12 participants), classroom-plus-simulation intervention (16 participants), or control condition (19 participants); ~4 members per team) | General teamwork intervention | 3: general teamwork | **Classroom-only**: *Crew Resource Management (CRM)* training in one-day course involving facilitated discussion, case studies, video vignettes, role-plays, and practice of teamwork. **Simulation-only**: *CRM*-style one-day training, addressing the same competencies as classroom-only training by participating in and observing scenarios in the patient simulation facility. **Classroom-plus-simulation**: included both classroom and simulation training described above, two weeks apart**. Control**: No classroom or simulation training. |
| Deneckere 2013 [46] | Intact: orthopedic surgeons, pneumologists, head nurses, nurses, physiotherapists, and social workers from 30 hospitals (17 intervention, 346 members; 13 control, 235 members); ~20 members per intervention team | Preparation (vision), execution (coordination, communication), reflection (feedback, innovation), interpersonal dynamics (conflict management) | 3: coordination, innovation, conflict management | **Experimental**: Workshop training in care pathways development. **Control**: No training. |
| Emmert 2011 [49] | New: health profession students (24 intervention participants; 22 control participants) | General teamwork intervention | 3: general teamwork | **Experimental**: Three-hour course on applying teamwork skills to the geriatric population. **Control**: Three-hour attention control course that was unrelated to teamwork. |
| Jankouskas 2010 [7] | New: nursing and medical students (12 intervention participants, 12 control participants); 4 members per team | Preparation (goal setting, plan), execution (communication, coordination), reflection (situation awareness, performance monitoring, backing up, problem solving) | 7: performance (4), communication, situation monitoring, general teamwork | **Experimental**: *Crew Resource Management (CRM)* program: didactic presentation of team processes, videotaped scenario practice using human patient simulator, and instructor-led group reflection of simulation. Training sessions lasted 3 hours. **Control**: Participants received a review of basic life support skills. |
| Kim 2014 [53] | Intact: nurses and physicians from two healthcare organizations (1 intervention, 25 members; 1 control, 36 members) | Preparation (mission analysis, goal setting, planning), execution (cooperation, communication), reflection (feedback, performance monitoring, situational awareness, problem solving, backing up), interpersonal dynamics (conflict management) | 4: communication (2), general teamwork (2) | **Experimental**: *TeamSTEPPS* program consisted of one 1.5-hour education session; one 10-minute simulation, based on class concepts with a 20-minute debriefing session; and a 2-hour CD consisting of modules and video vignettes. **Control**: No training. |
| Marshall 2009 [22] | New: medical students in 17 teams (8 intervention, 83 members; 9 control, 85 members) | Preparation (planning), execution (communication, coordination), reflection (problem solving, intrateam coaching, feedback) | 1: communication | **Experimental**: *ISBAR (Identify, Situation, Background, Assessment, Recommendation)* program consisted of 40-minute, small-group teaching session consisting of group discussions on the importance of communication, critique of videos showing suboptimal communication, introduction of *ISBAR* tool, video of *ISBAR* in use, and paper-based scenarios and role plays of students practising the use of the tool. **Control**: No training. |
| Morey 2002 [3] | Intact: emergency department physicians, nurses, and technicians from nine hospitals (6 intervention hospitals [3 military, 3 civilian], 684 members; 3 control hospitals [1 military, 2 civilian], 374 members); ~114 members per intervention team | Preparation (planning), execution (communication, cooperation, coordination), reflection (problem solving, situational awareness), interpersonal dynamics (conflict management) | 2: Performance, general teamwork | **Experimental**: Eight-hour workshops consisting of approximately 16 participants per session. Included videos of good and poor teamwork, practical team exercises for participants to practice teamwork (e.g., task prioritization, case review from a teamwork perspective), and analysis/discussion of clinical vignettes conveying good and poor teamwork. **Control**: Delayed/waitlist control—participants received training after experimental period. |
| O’Leary 2011 [21] | Intact: two hospitals (1 intervention, 81 members; 1 control, 66 members) | Preparation (planning), execution (communication) | 1: general teamwork | **Experimental**: *Structured Interdisciplinary Rounds (SIDR)* with regular interdisciplinary meetings included daily goals of care forms. Meetings occurred each weekday for 30-40 minutes. **Control**: No training. |
| Shapiro 2004  [57] | Intact: emergency department personnel comprising four teams (2 intervention teams, 8 participants; 2 control teams, 8 participants) | Execution (communication, cooperation, coordination), reflection (feedback) | 1: general teamwork | **Experimental**: One-day session consisted of an overview of crew resource management followed by three simulations of patient care (approximately 30 minutes each). Debriefs followed each simulation. **Control**: No training. |
| Thomas 2007 [58] | New: medical interns in pediatrics, internal medicine & pediatrics, family medicine, and obstetrics and gynecology (17 intervention participants; 15 control participants) | Preparation (planning), execution (communication, cooperation), reflection (performance monitoring, situational awareness) | 1: general teamwork | **Experimental**: Participants received Neonatal Resuscitation Program and 2.5-hour team training program included lectures, role-play simulations, video clips, and question & answer period designed to illustrate effective teamwork behaviors. **Control**: Participants received usual Neonatal Resuscitation Program with no team training. |
| Weaver 2010 [60] | Intact: surgeons, nurse anesthetists, nurses, surgical technicians, anesthesiologists, and physician assistants from two hospitals (1 intervention, 29 members; 1 control, 26 members) | Preparation (mission analysis, goal setting, planning), execution (cooperation, communication), reflection (feedback, performance monitoring, situational awareness, problem solving, backing up), interpersonal dynamics (conflict management) | 6: general preparation, planning, communication (2), situation monitoring, social support | **Experimental**: *TeamSTEPPS* program consisted of four-hour training session, including role-playing activities. **Control**: No training. |
| Weller 2014 [61] | Intact: 40 anesthetists from two hospitals, working with a nurse and anesthetic technician (i.e., 3 members per team) completed two crisis interventions (one under experimental condition and one under control condition) | Preparation (planning), execution (communication), reflection (problem solving, situational awareness) | 2: communication, general teamwork | **Experimental**: Anesthetists viewed a video demonstrating the use of an acronym within a simulation designed to improve team communication. **Control**: Anesthetists viewed an attention control video (discussing a separate, irrelevant acronym). |
| **LABORATORY EXPERIMENT** | |  | |  |
| Bjornberg 2014 [9] | New: undergraduate students comprising 47 virtual teams (80 experimental participants, 80 control participants); 3-4 members per team | Reflection (mutual performance monitoring) | 6: performance (4), performance monitoring (2) | **Experimental**: 120-minute experimental session, included training on effective mutual team member performance monitoring. **Control**: Completed same 120-minute experimental session but did not receive training on performance monitoring. |
| Brown 2003 [62] | New: undergraduate business students comprising 42 teams (21 experimental teams, 92 members; 21 control teams, 92 members); ~4 members per team | Execution (communication), reflection (problem solving, intrateam coaching) | 1: performance | **Experimental**: 75-minute training session consisting of a discussion of the importance of teams and teamwork in organizations, and the impact of self and others-statements on team performance. Participants were then trained to change dysfunctional/negative statements to functional/ positive ones that guide behavior. A 30-minute follow-up session reviewing principles of the training session occurred five weeks later. **Control**: 75-minute session wherein participants had a general discussion of the importance of teams and teamwork |
| Dibble 2010 [47] | New: undergraduate/graduate business students assigned to external adjustment intervention, placebo training, or no training control condition; ~5-6 members per team | Reflection (systems monitoring, problem solving, innovation) | 2: performance, general reflection | **Experimental**: Discussion of various types of external challenges teams might possibly encounter in the experimental team task, a range of adjustment strategies that could be utilized, examples of each type of adjustment strategy, a review of the risks of over- and under-adjusting, and guidelines for selecting an appropriate adjustment strategy. **Control**: No training. |
| Ellis 2005 [14] | New: students from management course (31 intervention teams, 124 members; 34 control teams, 136 members); 4 members per team | Preparation (planning), execution (coordination, communication), reflection (problem solving) | 3: planning, communication, problem solving | **Experimental**: 30-minute training session consisted of didactic lecture included nine case studies highlighting critical aspects of teamwork, reflection on each, and developing plans of action (with feedback from instructors on correct plans). **Control**: No training. |
| Haslam 2009 (study 1) [67] | New: undergraduate students assigned to imposed goal setting condition (9 teams, 34 participants), participative goal setting condition (9 teams, 34 participants), or control condition (9 teams, 32 participants) | Preparation (action planning) | 4: for both conditions: performance (2) | **Imposed goal setting**: participants given goal of improving performance by 20% in phase two and 40% in phase three of the experiment compared to their baseline performance. **Participative goal setting**: participants given same targets as participants in the imposed goal setting condition, but told to reflect on this target and set a goal themselves (in both phase two and phase three). **Control**: No goal setting; told to do their best |
| Haslam 2009 (study 2) [67] | New: undergraduate students assigned to imposed-easy goal condition (12 teams, 41 participants), imposed-difficult goal condition (15 teams, 50 participants), participative-easy goal condition (13 teams, 38 participants), participative-difficult goal condition (15 teams, 47), or control condition (16 teams, 50 participants) | Preparation (action planning) | 8: for each condition: performance (2) | **Imposed-easy goal setting**: participants given goal of improving performance by 20% in phase two and 40% in phase three of the experiment compared to their baseline performance. **Imposed-difficult goal setting**: participants given goal of improving performance by 40% in phase two and 80% in phase three of the experiment compared to their baseline performance. **Participative-easy goal setting**: participants given same targets as participants in the imposed-easy goal setting condition, but told to reflect on this target and set a goal themselves (in both phase two and phase three). **Participative-difficult goal setting**: participants given same targets as participants in the imposed-difficult goal setting condition, but told to reflect on this target and set a goal themselves (in both phase two and phase three)**. Control**: No goal setting; told to do their best |
| Jarrett 2012 [69] | New: undergraduate psychology students assigned to local/ subjective after-action review (AAR) intervention (20 teams, 80 participants), local/objective AAR intervention (20 teams, 80 participants), local control condition (23 teams, 92 participants) distributed/subjective AAR intervention (20 teams, 80 participants), distributed/objective AAR intervention (20 teams, 80 participants), or distributed control condition (20 teams, 80 participants); 4 members per team | Preparation (mission analysis, goal setting), reflection (feedback, performance monitoring, problem solving) | 4: for both conditions: performance | **Subjective AAR**: Members participated in 10-minute review (monitored by experimenter) of performance on experimental team task, identify whether goal had been met, identify behaviors that affected the attainment of mission outcomes, and set outcome and behavioral goals for subsequent experimental team tasks. For the distributed condition, members were in separate locations; for the local condition, members in the same location. **Objective AAR**: Members participated in same review as that described above but also had the ability to use the video simulator as they reviewed their previous performance. For the distributed condition, members were in separate locations; for the local condition, members in the same location. **Control**: Members completed a filler task that was unrelated to team task. |
| Kring 2005 [70] | New: undergraduate/graduate students assigned to local team communication training (TCT) intervention (16 participants), distributed TCT intervention (16 participants), local control condition (16 participants), or distributed control condition (16 participants); 2 members per team | Execution (communication), reflection (feedback, performance monitoring, problem solving) | 10; for both conditions: performance | **Experimental**: 1-hour session, wherein participants read four short descriptions of communication dimensions (process, information exchange, feedback, and shared models) and then practiced the main parts of each while completing a collective task with the experimenter. **Control**: Participants completed a filler task unrelated to teamwork |
| Martinez-Moreno 2015 [54] | New: undergraduate psychology students assigned to intervention (28 teams, 112 members) or control condition (26 control teams, 104 members); 4 members per team | Reflection (performance monitoring, feedback), Interpersonal dynamics (conflict management) | 7: backing up, conflict management (6) | **Experimental**: Team self-guided training: instructor-led review of team processes and outcomes on experimental task, and how team functioning could be improved in the second experimental task. **Control**: No training. |
| Prichard 2007 [55] | New: undergraduate students assigned to intervention (4 teams, 24 participants) or control condition (4 teams, 24 participants) | Planning (goal setting, action planning), execution, reflection (performance monitoring, problem solving, feedback), interpersonal dynamics (conflict management) | 7: coordination, communication, planning, problem solving, situational monitoring, general teamwork (2) | **Experimental**: Members given 90-minute Chalybeate Team Development in Universities programme, wherein they are trained on various team skills (e.g., problem solving, action planning, interpersonal relations). **Control**: No training. |
| Schurig 2013 [72] | New: undergraduate psychology students assigned to subjective after-action review (AAR) intervention (40 teams, 160 participants), objective AAR (40 teams, 160 participants), or control condition (43 teams, 172 participants); 4 members per team | Preparation (planning), reflection (performance monitoring, feedback) | 2; for both conditions: performance | **Subjective AAR**: Members participated in 10-minute review of performance on experimental team task, identify whether goal had been met, identify behaviors that affected the attainment of mission outcomes, and set outcome and behavioral goals for subsequent experimental team tasks. **Objective AAR**: Members participated in same review as that described above but also had the ability to use the video simulator as they reviewed their previous performance. **Control**: Members completed a filler task that was unrelated to team task. |
| Smith-Jentsch (study three) 1996 [61] | New: undergraduate psychology students assigned to lecture-only intervention (15 participants), behavioral role-modeling intervention (15 participants), lecture-plus-demonstration intervention (15 participants), or control condition (15 participants); two members per team | Interpersonal dynamics (conflict management) | 3; for all three conditions: communication | **Behavioral role-modeling**: 10-minute lecture about team performance-related assertiveness, persuasive arguments, and encouragement for using this strategy in team environments; participants then reviewed videos of role models demonstrating various responses to conflict as well as feedback from experimenter about the scenes. **Lecture-plus-demonstration**: Same 10-minute lecture and video scenes described above, with the addition of the experimenter reviewing relevant learning points from the lecture before and after each scene. **Lecture-only**: Expanded 1-hour lecture of the same lecture described above. **Control**: No training. |
| Villado 2013 [16] | New: undergraduate psychology students assigned to subjective after-action review (AAR) intervention (11 teams, 44 members), objective after-action review (12 teams, 48 members) intervention, or control condition (24 teams, 96 members); 4 members per team | Preparation (mission analysis, goal setting, planning), reflection (feedback, performance monitoring, problem solving) | 2; for both conditions: performance | **Subjective AAR**: Team members received mission feedback and completed a 10-minute review of their performance on the experimental task, identify whether goal had been met, identify behaviors that affected the attainment of mission outcomes, and set outcome and behavioral goals for subsequent experimental team tasks. **Objective AAR**: Members received mission feedback participated in same review as that described above but also had the ability to use the video simulator as they reviewed their previous performance. **Control**: Members received mission feedback but no after-action review |
| Volpe 1996 [59] | New: undergraduate students assigned to cross-training intervention (20 teams, 40 participants) or control condition (20 teams, 40 participants); 2 members per team | General teamwork intervention | 6: general teamwork, communication (5) | **Experimental**: Participants informed about the operationally relevant tasks pertaining to their own functional responsibilities as well as those of the other position. Training lasted 30-45 minutes. **Control**: Participants only informed about the tasks pertaining to their own responsibilities. |
| Wegge 2005 [77] | New: students assigned to directive group goal setting (30 teams, 120 participants), participative group goal setting (30 teams, 120 participants), participative group plus individual goal setting (30, 120 participants teams), or control condition (30 teams, 120 participants); 4 members per team | Preparation (goal setting) | 3; for each condition: performance | **Directive group goal setting**: Members given a specific target to strive towards. **Participative group goal setting**: Members instructed to set challenging group goal. **Participative group plus individual goal setting**: Members instructed to set challenging group goal as well as their own individual goal. **Control**: Members instructed to do their best. |
| **ACADEMIA** | |  |  |  |
| Aaron 2014 [13] | New: 88 undergraduate students assigned to team charter intervention (28 students), team charter plus training, support, instruction intervention (29 students), or control condition (31 students) | Preparation (team charter), interpersonal dynamics (conflict management) | 4: communication (2), social support (2) | **Team charter**: Students created a team charter which included a mission statement, team norms, team goals, and performance measurement. **Team charter plus training, support, instruction**: Same as above, but students also received training, support, and instruction on utilizing the charter from the course instructor. **Control**: No team charter or training given. |
| Beck-Jones 2004 [41] | New: business students assigned to roles plus cross-training intervention (54), roles only intervention (52), or control condition (55); 4 members per team | Execution (coordination, cooperation) | 10; for both conditions: performance (2), goal setting, coordination, communication | **Roles only**: Students assigned roles and responsibilities to completing the assigned task. **Roles plus cross-training**: Students assigned roles and responsibilities and received information on the roles and responsibilities of the other team members. **Control**: No training. |
| Becker 2005 [40] | New: occupational therapy, physical therapy, and respiratory care students enrolled in an online course (48 intervention students, 47 control students) | Execution (communication), reflection (feedback, problem solving), interpersonal dynamics (social support) | 1: general teamwork | **Experimental**: Students received feedback on team members’ interactions; module coordinators also provided weekly feedback to faculty mentors on methods to improve student interaction. **Control**: No feedback given. |
| Beranek 2005 [42] | New: undergraduate computer architecture students comprising 23 virtual teams (12 intervention teams, 36 members; 11 control teams, 33 members); 3 members per team | Preparation (goal setting), execution (communication), interpersonal dynamics (conflict management) | 1: general teamwork | **Experimental**: Students given training on effective teamwork, possible drawbacks to electronic communication, and common “ebbreviations” to assist with virtual communication. **Control**: No training. |
| Bushe 1995 [43] | New: undergraduate students assigned to appreciative inquiry (32 members), team development (32 members), or control (32 members) condition; 4 members per team | Preparation (goal setting, action planning), reflection (performance monitoring, feedback) | 6; for both conditions: performance, conflict management, problem solving | **Appreciative Inquiry**: Members each reflect on their best experience in a team, then come up with a list of what a highly effective team looks like. **Team Development**: Members reflect on the team’s processes, and then develops goals and action plans  **Control**: No training. |
| Fandt 1990 [66] | New: business course students (89 intervention students; 43 control students) | Preparation (goal setting, planning); execution (communication) | 1: performance | **Experimental**: Participants given a lecture and assignment on goal setting for a team simulation task. Goals were taught to be moderately difficult, specific, concrete, and measurable. **Control**: No training. |
| Padmo Putri 2012 [6] | Intact: faculty members, instructional designer, and course manager from four departments of a university (32 intervention teams, 71 members; 33 control teams, 75 members); 3 members per team | Preparation (goal setting, planning), execution (communication) | 7: performance (3), planning (2), communication (2) | **Experimental**: 1.5-hour training consisted of strategies for communication and planning during course development workshop. Participants viewed a presentation on developing a communication strategy plan, and practiced creating a plan. **Control**: Participants given a presentation unrelated to teamwork/communication training. |
| Rapp 2007 [56] | New: Students from two sections of a course assigned to experimental (26 students) or control (28 students) condition | Preparation (mission analysis, goal setting, action planning), execution (communication, coordination), reflection (feedback, problem solving), interpersonal dynamics (conflict management) | 2: performance, general teamwork | **Experimental**: Members given CD-based training program comprised of 13 modules related to managing teamwork (e.g., developing team charters, managing conflict, goal setting, communication). **Control**: No training. |
| Sikorski 2012 [74] | New: undergraduate meteorology students (29 intervention teams, 99 students; 30 control teams, 102 students) | Preparation (planning), execution (communication, coordination), reflection (innovation, problem solving) | 3: performance | **Experimental**: Members rate team on team and task-related knowledge factors, and then identify two areas that can be improved and plans for how these areas can be improved. **Control**: Participants completed a newspaper reading task, unrelated teamwork. |
| **AVIATION** |  |  |  |  |
| Brannick 2005 [5] | New: pilots assigned to intervention (24 teams, 48 members) or control condition (24 teams, 48 members); 2 members per team | Preparation (planning), execution (coordination, communication), reflection (feedback, performance monitoring, problem solving, intrateam coaching) | 5: performance (3), coordination (2) | **Experimental***: Crew Resource Management* training consisted of lecture on communication, assertiveness, and situation awareness. Participants then shown a short film of aircrews demonstrating these three skills during flight scenarios. Discussion of the lecture and film then occurred. Feedback from experimenter for the teams on their simulation performance also provided. **Control**: Participants completed problem-solving exercises and video games unrelated to teamwork. |
| Ikomi 1999 [68] | Intact: airline crews assigned to experimental (27 crews) or control condition (20 crews) | Execution (communication, coordination) | 3: performance | **Experimental**: Crew resource management principles designed to enhance coordination and communication embedded within in-situ flight procedures. **Control**: No training. |
| Siegel 1973 [73] | Intact: helicopter pilots and navigators assigned to intervention (8 teams, 16 members) or control (8 teams, 16 members) condition; 2 members per team | Execution (communication) | 1: performance | **Experimental**: Helicopter teams observed a team completing a flight simulation that incurred a flight problem and listened to the team’s communication. After each problem, all teams would reconvene to discuss various questions. **Control**: No training. |
| Stout 1997 [76] | New: aviators assigned to intervention (20 participants) or control condition (22 participants) | Preparation (planning), Execution (communication, coordination), reflection (situational awareness, problem solving), interpersonal dynamics (conflict management) | 3: performance | **Experimental**: Over two days, participants were educated on communication, assertiveness, and situational awareness via lectures, demonstrations (videotapes and case study analyses) of teamwork concepts, and practice and feedback administered by means of role-plays and simulator exercise (which focused on the three teamwork skills). **Control**: Delayed/waitlist control—participants received training after experimental period. |
| **MILITARY** | |  |  |  |
| Cannon-Bowers 1998 [64] | New: Navy recruits assigned to experimental (20 teams, 60 participants) or control (20 teams, 60 participants) condition; 3 members per team | Execution (coordination, communication, cooperation) | 3: performance | **Experimental**: Members instructed on their job duties/roles as well as those of their teammates. **Control**: Members only instructed on their own responsibilities. |
| Dalenberg 2009 [45] | New: cadets assigned to intervention (22 teams, 88 members) or control condition (19 teams, 76 members); 4 members per team | Preparation (goal setting, planning), execution (cooperation, coordination, communication) | 3: performance, coordination, general teamwork | **Experimental**: Participants instructed to develop plans with consideration for the following: setting team goals, prioritizing, defining roles, passing information, coordination of action, cooperation, and assistance. Discussions took up to ten minutes. **Control**: No discussion time given. |
| Eden 1986 [48] | Intact: military company members assigned to experimental (7 companies, 220 members) or control (9 companies, 280 members) condition | General teamwork intervention | 2: general teamwork, conflict management | **Experimental**: Three-day workshop on teamwork development. **Control**: No training. |
| Entin 1999 [50] | New: naval officers assigned to team adaptation and coordination training (TACT) intervention (2 teams, 10 members), TACT+ intervention (2 teams, 10 members), or control condition (2 teams, 10 members); 5 members per team | Preparation (planning), execution (coordination, communication), reflection (feedback, performance monitoring, systems monitoring, situational awareness, backing up), interpersonal dynamics (social support) | 2: performance, coordination | **TACT**: Teaches teams coordination and communication strategies, including: (1) how to identify signs and symptoms of stress in the external environment, in the team, and in individual members; (2) instructions on, and videos of, five adaptive strategies that can be used to cope with increased workload and stress; and (3) practicing strategies in two 12-minute training scenarios, with feedback on teamwork behaviors. **TACT+**: Same as above, plus tactical action officer of the team was given specific instructions and practice on how to give brief (~30 second) situation-assessment updates (current priorities, targets of interest, and situation perception) to the rest of the team (approximately once every 3 minutes). **Control**: members told they were being trained to appreciate the “big picture” and how their team’s performance affected other teams on their platform and in the battle group |
| Green 1994 [52] | New: Naval students assigned tactical adaptation (TACT) (10), tactical adaptation and coordination (TACT+) (10), or control (10) condition | Execution (coordination) for TACT; Execution (coordination), reflection (situational monitoring) for TACT+ | 4; for both conditions: performance, general teamwork | **TACT**: Teams taught teamwork strategies (focused on coordination) in adapting to stressful military situations **TACT+**: Same training as above, and also prompted to provide situation reports every three minutes. **Control**: No training. |
| Smith-Jentsch 2008 [75] | Intact: navy teams assigned to intervention (7 teams, 35 members) or control condition (6 teams, 30 members); 5 members per team | Preparation (mission analysis), reflection (problem solving, performance monitoring, feedback) | 4: performance, communication, cooperation, general teamwork | **Experimental**: Team leaders given training for two hours on how to facilitate guided team self-correction. Then, after team task simulations, leader had team identify positive and negative instances of teamwork behaviors. Leader then asked the team to generate four specific goals for improvement on the next team task. **Control**: Team leaders received taskwork-related training. |
| **INDUSTRY** |  |  |  |  |
| Buller 1986 [63] | Intact: hard rock miner teams assigned to teambuilding (6 teams, 18 members), goal setting (4 teams, 8 members), teambuilding plus goal setting (6 teams, 18 members), or control (4 teams, 9 members) condition | Reflection (feedback, performance monitoring) for teambuilding condition; Preparation (goal setting, action planning), reflection (feedback, performance monitoring) for goal setting condition, and teambuilding plus goal setting condition | 9: for each condition, performance (3) | **Teambuilding**: Teams reflect on team processes, guided by three questions: ‘how can we do our job better?’, ‘how can we make this a better place to work?’, and ‘how can we make this a safer place to work?’. **Goal setting**: Teams received feedback on their current performance and then set specific, challenging performance goals for the following three months. Feedback on progression to goal attainment were then given each week for the next three months**. Teambuilding plus goal setting**: Combination of the above two conditions. **Control**: No training |
| Friedlander 1967 [51] | Intact: employees from a research and development organization assigned to intervention (4 teams, 31 members) or control (8 teams, 60 members) | Preparation (action planning), reflection (problem solving) | 1: general teamwork | **Experimental**: Team members identify problems facing the team, brainstorm possible solutions to these problems, and plan how these solutions can be implemented. **Control**: No training. |
| Longenecker 1994 [71] | Intact: manufacturing teams assigned to intervention (26 members) or control (26 members) condition | Preparation (goal setting), reflection (feedback, problem solving) | 1: performance | **Experimental**: Teams set specific, challenging performance goals. Performance was monitored for each shift, and reviewed/revised as necessary. **Control**: No training. |
